# Molecular characterization of *Rhodococcus equi* isolates from horses in Poland: pVapA characteristics and plasmid new variant, 85-kb type V

**DOI:** 10.1186/s12917-017-0954-2

**Published:** 2017-01-26

**Authors:** Lucjan Witkowski, Magdalena Rzewuska, Shinji Takai, Dorota Chrobak-Chmiel, Magdalena Kizerwetter-Świda, Małgorzata Feret, Marta Gawryś, Maciej Witkowski, Jerzy Kita

**Affiliations:** 1 Laboratory of Veterinary Epidemiology and Economics, Faculty of Veterinary Medicine, Warsaw University of Life Sciences, Nowoursynowska 159c, 02-776 Warsaw, Poland; 2 Department of Preclinical Sciences, Faculty of Veterinary Medicine, Warsaw University of Life Sciences, Ciszewskiego 8, 02-786 Warsaw, Poland; 3 Department of Animal Hygiene, School of Veterinary Medicine, Kitasato University, Higashi 23-35-1, Towada, Aomori 034-8628 Japan; 4 University Center of Veterinary Medicine UJ-UR, Mickiewicza 24/28, 30-059 Cracow, Poland

**Keywords:** Rhodococcosis, Epidemiology, PFGE, Horse, Plasmid profile

## Abstract

**Background:**

*Rhodococcus equi* is one of the most significant bacterial pathogens affecting foals up to 6 months of age worldwide. Rhodococcosis is present in Poland however information about molecular characterization of *R. equi* isolates is scarce.

This study describes molecular characterization of *Rhodococcus equi* infection on 13 horse breeding farms in Poland between 2001 and 2012. Samples were collected by tracheobronchial aspiration from pneumonic foals or during necropsy. The *R. equi* isolates were genotyped by plasmid profiling and pulsed-field gel electrophoresis.

**Results:**

Totally, 58 *R. equi* isolates were investigated. One isolate lost its plasmid. Among the 57 VapA-positive isolates, 48 contained 85-kb type I plasmid (82.8%), 8 contained 87-kb type I plasmid (13.8%). One isolate (1.7%) had a unique restriction cleavage pattern and the 2nd fragment of *Eco*RI digests of this plasmid DNA was about 2600 bases smaller than that of the 85 kb type I. This new plasmid variant was designated as the “85-kb type V”.

Among the 58 isolates typeable with *Vsp*I-PFGE, ten PFGE clusters were detected. The majority of foals were infected mostly with isolates of low genetic diversity.

**Conclusions:**

Most of clinical isolates of *R. equi* from foals in Poland contain pVapA 85-kb type I and 87-kb type I similarly to the other European countries and the United States. However, the new variant of pVapA 85-kb type V was identified.

The chromosomal variability was detected among some of the investigated isolates and the presence of farm-specific isolates might be possible.

## Background

*Rhodococcus equi* is one of the most significant bacterial pathogens affecting foals up to six months of age and occasionally can cause diseases in other animal species including humans [1–6]. Typical manifestation of the infection in foals includes pyogranulomatous bronchopneumonia with abscessation. Epidemiology of the disease is similar worldwide: it occurs endemically on some farms and rarely strikes the others. The prevalence and severity of the disease on endemic farms may vary among seasons [1–5]. *R. equi* infection in foals has been recognized and described in Poland [7–9], but without any information about molecular characterization of *R. equi* isolates. Recently, prevalence of *R. equi* in horse carcasses intended for human consumption was investigated in Poland. Single *R. equi* strain carried 85-kb type I plasmid was isolated from 0.5% (1/198) samples of healthy middle tracheo-branchiales lymph nodes while no lymphocentrum retropharyngeum sample was positive (0/198) [10].

The virulence of *R. equi* is determined by genes *vaps* located on virulence-associated plasmids (VAPs). Isolates carrying the *vapA* gene encoding virulence-associated 15–17-kDa protein (VapA) are known as virulent and are capable of causing disease in foals [11, 12]. To date analysis of the restriction enzyme digestion patterns revealed 12 distinct VAPA types and some of them have geographical specificity [13–24].

Epidemiological studies on genetic relations among *R. equi* isolates are usually based on genotyping by the pulsed-field gel electrophoresis (PFGE) method. Some of the studies report that horses from individual regions or farms seem to harbor isolates with low genetic diversity [13, 23, 25]. On the other side, the great diversity in the genotypes of isolates within and among farms or among countries also has been shown [26]. Furthermore, one foal can be infected with various *R. equi* isolates that vary in plasmid type and genotype [17, 27].

The aim of this study was to characterize types of virulence plasmids in isolates originating from Polish farms and to determine the genetic diversity of the isolates.

## Results

**Fig. 1:**
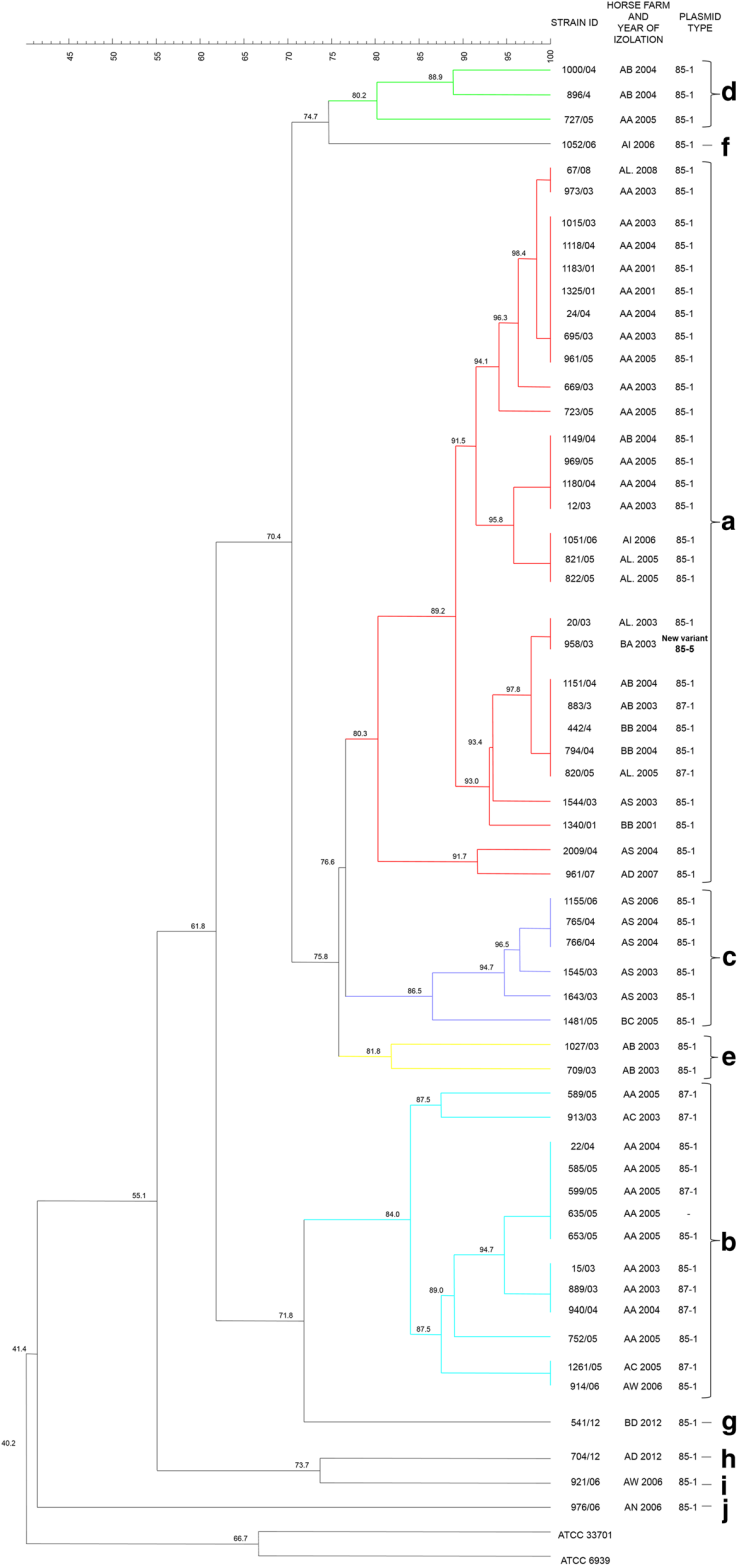
Dendrogram of 57 virulent and 1 plasmid cured isolates of *R. equi* obtained from foals from Poland genotyped with *Vsp*I-PFGE method. Among the isolates ten PFGE clusters were detected clusters (**a**-**j**)

Totally 58 isolates from 13 horse farms were investigated. The isolate ID (code of the horse farm) and year of isolation are presented in Fig. 1. The *vapA* gene specific for *R. equi* which is virulent for horses was detected in all 58 isolates. However, the virulent VapA-positive isolate 635/05 lost its plasmid during subculturing. Thus, PFGE was performed on 58 whereas plasmid profiling on 57 isolates.

**Fig. 2:**
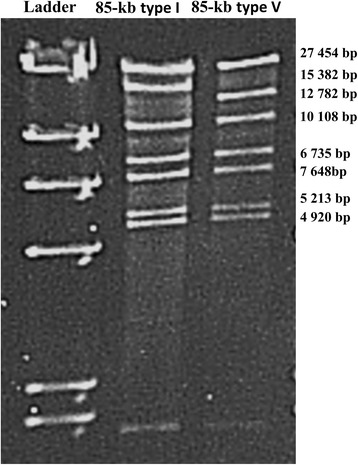
EcoRI restriction fragments of the plasmids of *R. equi*. Lanes: 1, 85-kb type I; line 2, 85-kb type V (new type)

Among 57 VapA-positive isolates, 48 (82.8%) contained 85-kb type I plasmid, 8 (13.8%) contained 87-kb type I plasmid (Table 1). One isolate (1.7%) had a unique restriction cleavage pattern and the 2nd fragment of *Eco*RI digests of this plasmid DNA was about 2600 bases smaller than that of the 85 kb type I (Fig. 2). This new plasmid variant was designated as the “85-kb type V”.

**Table 1:** Plasmid profiles of VapA-positive *R. equi*

No.	Plasmid type	Isolation rate (%)	Sample source	Country	Reference
1.	85-kb type I	96.4%	Foal	Germany	[13]
		95.3%	Foal	Hungary	[15]
		82.8%	Foal	Poland	This study
		71.4%	Soil	Hungary	[20]
		69.0%	Foal	France	[14]
		64.5%	Soil	USA	[16]
		54.2%	Soil	Hungary	[15]
		53.1%	Foal	USA	[16]
		41.7%	Soil and feces	France	[14]
		38.5%	Foals	Argentina	[28]
		14.6%	Foal	Brazil	[19]
		14.6%	Foals	Brazil	[19]
		3.6%	Soil	South Africa	[18]
		-	Foals	South Africa	[18]
		100%	Adult horse lymph node	Poland	[10]
2.	85-kb type II	4.9%	Foals	France	[14]
3.	85-kb type III	15,3%	Soil	Hungary	[20]
		8.0%	Soil	USA	[16]
		4.2%	Foals	USA	[16]
4.	85-kb type IV	2.1%	Foals	USA	[16]
		1.1%	Soil	USA	[16]
5.	85-kb type V	1.7%	Foal	Poland	This study
6.	87-kb type I	80.5%	Foals	Brazil	[19]
		80.0%	Foals	Brazil	[19]
		57.7%	Foals	Argentina	[28]
		40.6%	Foals	USA	[16]
		27.8%	Soil and feces	France	[14]
		26.4%	Soil	USA	[16]
		24.6%	Foals	France	[14]
		13.8%	Foal	Poland	This study
		13.3%	Soil	Hungary	[15]
		7.1%	Soil	South Africa	[18]
		4.7%	Foals	Hungary	[15]
		3.6%	Foal	Germany	[13]
7.	87-kb type II	85.2	Foals	Japan	[28]
8.	87-kb type III	4.9%	Foals	Brazil	[19]
9.	90-kb type I	74.5%	Foals and dams	Japan	[22]
10.	90-kb type II	47.1%	Foals, soil and feces	Korea	[23]
		0.8%	Soil and feces	Korea	[24]
11.	90-kb type III	24.7%	Foals and dams feces	Japan	[22]
		-	Foals	Japan	[14]
12.	90-kb type IV	0.9%	Foals and dams feces	Japan	[22]
		-	Foals	Japan	[14]
13.	90-kb type V	52.9%	Foals, soil and feces	Korea	[23]
		1.2%	Soil and feces	Korea	[24]

All 58 VapA-positive isolates were typeable with *Vsp*I-PFGE, resulting in the detection of ten PFGE clusters (from A to J) (Fig. 1). The major cluster (A) was shared by 29 isolates from 8 horse farms. Cluster B was shared by 13 isolates from 3 farms, cluster C by 6 isolates from 2 farms. Four isolates from 2 farms belonged to cluster D and 2 isolates from one farm belonged to cluster E. The remaining 4 isolates exhibited various pulsed-field patterns. The most of investigated isolates belonged to three main clusters (A. B and C). Part of them were indistinguishable and had the same PFGE pattern despite the fact that they were isolated on various horse farms in various years. PFGE patterns of the isolates were compared with virulence plasmid types and no concordance was observed. Some of the isolates carried pVapA 85-kb type I, 87-kb type I, new variant 85-kb type V, as well as the one had lost the plasmid had the same PFGE pattern.

## Discussion

The prevalence of plasmid 85-kb type I and 87-kb type I in Poland is similar to other studies from Europe [13–15] as well as the USA [16], while in the South America 87-kb type I is most often isolated than 85-kb type I [19, 28]. New type of plasmid 85-kb type V might be a local type similar for example to 85-kb type IV in Texas [16] (Table 1).

We were not able to investigate plasmid type in one case because of the plasmid loss [11, 14, 29]. The guidelines for molecular epidemiologic investigation using PFGE assay indicate that the isolate characterization should only be applied to small sets of isolates related to potential outbreaks [30]. In this study, investigated isolates met these criteria as the breeding horse population in Poland is relatively small and mostly concentrated on several breeding farms and most of the isolates used in this study were collected in several years during *R. equi* outbreaks.

The study has several limitations. Firstly, it was a descriptive study: we used the isolates obtained from collaborators as diagnostic material (no random sampling). Secondly, samples were obtained from all of the suspected horses only from a few farms and only during one or a few seasons (farms AA and AB). That’s the reason why the isolates from those farms are overrepresented in the analysis. Most of the isolates from the other farms were isolated from individual samples delivered to the laboratory. No data concerning the number of suspected or affected foals on a farm were available. In conclusion, collected isolates and data were not sufficient for the proper statistical analysis and obtained results are only descriptive.

Our results are consistent with some previous data [13, 23, 25] where a particular isolate or isolates with low genetic diversity are associated with the individual farms or regions. We identified the isolates typical for certain farms. For example almost all predominant isolates from the farm AA belonged to two main PFGE clusters (A and B) and most of them were indistinguishable. On the other hand, the same isolates occur on more than one farm, for example on the farms AI and AL as well as BB and AL. The high similarity of isolates on mentioned farms may be explained by the exchange of mares between them. Mares shed *R. equi* with feces in which the pathogen may multiply and inhalation of virulent *R. equi* is the major route of infection of foals in the first few days of life [3, 5]. Furthermore, the infected foals shed a large amount of VapA positive *R. equi* and this may explain why several major pulsotypes were widespread in the environment of endemic farms. Moreover, the analyzed samples were collected mostly during outbreaks (rhodococcosis morbidity in foals reached even 100%) at relatively close time intervals (year by year).

Comparison of PFGE patterns and plasmid type which was conducted in this study confirms previously indicated observations in horses [13], pigs [31] cattle [32] and wild boars [21]. However, *R. equi* isolates containing the same plasmid type revealed different PFGE patterns and isolates with identical PFGE patterns contained different virulence plasmids or were plasmid-less.

During the study we detected chromosomal variability among some of investigated isolates what had been previously described even among isolates collected from the same farm [26, 33, 34]. However, these studies were performed on a high number of isolates from heterogeneous horse populations with numerous clinical cases and soil samples from many farms contrary to our study conducted on smaller farms that stay in contact, for example by exchange of the horses.

Given the limitations of this study we can only speculate that the persistence of farm-specific isolates might be possible on some horse farms with limited movement of horses in Poland. Moreover, our observations are supported by reports from Germany and Australia were particular isolates were associated with individual farms [13, 25].

## Conclusions

Most clinical isolates of *R. equi* from foals in Poland contain VapA plasmids 85-kb type I and 87-kb type I similar to the other European countries and the United States. There is a new variant of VapA plasmid identified as 85-kb type V.

The chromosomal variability was detected among some investigated isolates, however farm-specific isolates appear to exist.

## Methods

Isolates of *R. equi* were obtained from samples collected by tracheobronchial aspiration from pneumonic foals or during necropsy. Foals originated from 13 horse breeding farms in Poland and were examined between 2001 and 2012.

Bacteria isolation, phenotypic and genotypic identification of isolates were conducted as described previously [35]. Briefly, modified CAZ-NB medium was used for bacteria isolation, API Coryne test (bioMerieux, France) was used for determination of biochemical properties and the presence of “equi factor” was studied in CAMP test. Presence of *R. equi* genes, *choE, traA* and *vapA* was determined by PCR. Alkaline lysis method with some modification was used for plasmid DNA isolation from the *vapA*-positive *R. equi* isolates. Isolated DNA was digested with restriction endonucleases EcoRI, EcoT221, HindIII and BamHI [35–38]. Bacterial DNA extraction and PFGE was performed as previously described [21, 23]. The *VspI* was used as the restriction enzyme. The gel images were analysed by Gel Compar II version 4.6 (Applied Maths, Belgium). Cluster analysis was performed by UPGMA using dice similarity coefficient with optimization set at 1% and position tolerance at 1.5%. The isolates were considered to be closely related and assigned to the same PFGE cluster using an 80% of homology cut-off [30]. The reference *R. equi* strains ATCC 6939 and ATCC 33701 were used as controls.

## Abbreviations

PFGE: Pulsed-field gel electrophoresis
VapA: Virulence-associated protein A
VAPs: Virulence-associated plasmids
